# Management of isocitrate dehydrogenase 1/2 mutated acute myeloid leukemia

**DOI:** 10.1038/s41375-024-02246-2

**Published:** 2024-04-10

**Authors:** Harry Fruchtman, Zachary M. Avigan, Julian A. Waksal, Nicole Brennan, John O. Mascarenhas

**Affiliations:** 1 New York Medical College, Valhalla, NY USA; 2 Tisch Cancer Institute, Icahn School of Medicine at Mount Sinai, New York, NY USA

**Keywords:** Drug development, Acute myeloid leukaemia

## Abstract

The emergence of next generation sequencing and widespread use of mutational profiling in acute myeloid leukemia (AML) has broadened our understanding of the heterogeneous molecular basis of the disease. Since genetic sequencing has become a standard practice, several driver mutations have been identified. Accordingly, novel targeted therapeutic agents have been developed and are now approved for the treatment of subsets of patients that carry mutations in *FLT3*, *IDH1*, and *IDH2* [1, 2]. The emergence of these novel agents in AML offers patients a new modality of therapy, and shifts treatment paradigms toward individualized medicine. In this review, we outline the role of *IDH* mutations in malignant transformation, focus in on a novel group of targeted therapeutic agents directed toward *IDH1*- and *IDH2*-mutant AML, and explore their impact on prognosis in patients with AML.

## Introduction

Acute myeloid leukemia (AML) is a hematologic malignancy originating at the level of the hematopoietic stem cell (HSC) with an overall incidence rate of 4.0 per 100,000 [3]. AML is primarily a disease of the elderly, with an age-adjusted incidence of 18.8 per 100,000 in patients >65 years compared to 4.9 per 100,000 in patients aged 50–64 [3]. Although the 5-year survival rate has tripled from 9% in 1980 to 27% in 2017, outcomes remain poor, particularly in older patients not eligible for intensive therapy [4]. The emergence of next generation sequencing and widespread use of mutational profiling has broadened our understanding of the heterogeneous molecular basis of AML [5]. Several targetable mutations in AML have been identified, and prior studies have shown the feasibility and efficacy of adding targeted therapies, such as FLT3 inhibitors, to improve responses both as part of initial therapy and after relapse [1, 2]. The emergence of novel agents targeting driver mutations in AML has offered patients a new modality of therapy and shifted treatments toward individualized medicine.

Mutations of isocitrate dehydrogenase (*IDH*) were first described in AML genome sequences in 2008 [5]. Mutations in *IDH* are seen in an estimated 14–20% of AML patients, with *IDH1* mutations occurring in 3–20% of cases and *IDH2* mutations in 9–20% of cases [6–9]. *IDH* mutations have been described in patients with other hematologic malignancies such as myelodysplastic syndrome (MDS) and myeloproliferative neoplasms (MPNs), highlighting the broad importance of IDH in the pathogenesis of myeloid malignancies [10]. Additionally, *IDH* mutations have been described in solid tumors such as gliomas, cholangiocarcinoma, chondrosarcoma, and lymphoid malignancies including T cell acute lymphoid leukemia [11–14].

The transformation of MPNs to AML, termed blast-phase MPN, confers a poorer prognosis compared to de novo AML. Several groups have demonstrated that *IDH* mutations are associated with MPN progression, particularly among patients with primary myelofibrosis (PMF) [15–17]. That is, the presence of *IDH1/2* mutations negatively impacts leukemia-free survival in PMF [18]. Those with MPN-blast phase with *IDH* mutations present may benefit from IDH inhibition therapy and should be studied in clinical trials.

In this review, we outline the role of *IDH* mutations in malignant transformation, focus in on a novel group of targeted therapeutic agents directed toward *IDH1*- and *IDH2*-mutant AML, and explore their impact on prognosis in patients with AML.

## Pathophysiology of *IDH* mutations in AML

Isocitrate dehydrogenases are a family of enzymes involved in metabolic and epigenetic processes. Isoforms IDH1 and IDH2 are NADP-dependent enzymes that catalyze the reversible oxidative decarboxylation reaction of isocitrate to α-ketoglutarate in the tricarboxylic acid cycle (TCA) and the reduction of NADP to NADPH, an important cofactor involved in the generation of adenosine triphosphate (ATP) in the electron transport chain (ETC) [19]. IDH1 and IDH2 are highly similar in structure, but IDH1 is found in the cytoplasm and peroxisome while IDH2 is predominantly found in the mitochondrial matrix [20]. Physiologically, IDH1 facilitates production of NADPH in the peroxisome, mainly for cholesterol synthesis [21]. Furthermore, IDH1 in the cytoplasm and peroxisome protect cells from oxidative damage by producing NADPH for the reduction of glutathione [22]. IDH2 primarily produces NADPH in the mitochondrial matrix for aerobic respiration [23].

Oncogenic mutations in *IDH1* and *IDH2* almost exclusively occur in functionally conserved arginine residues in the active site, specifically at R132 of *IDH1* and R140 or R172 of *IDH2* [24]. The R140 *IDH2* mutation occurs almost exclusively in myeloid malignancies, most commonly in AML, while alternate *IDH1* mutations are associated with several cancer types [24]. There is inconsistent data on the prognostic impact of specific IDH mutations, some studies suggest that R140 *IDH2* mutations are associated with a more favorable prognosis than R132 *IDH1* mutation or R172 *IDH2* mutation [25–27]. However, other studies demonstrate that there is no prognostic difference between R132 *IDH1*, R140 or R172 *IDH2* mutated AML [28]. R140 *IDH2* mutations are highly associated with nucleophosmin-1 (*NPM1*) mutations [26]. *NPM1*, one of the most commonly mutated genes in de novo AML, is mutated in 35% of patients and is associated with a favorable prognosis in newly diagnosed AML, but with a poor prognosis in relapsed or refractory (R/R) AML [28–31]. The association of R140 *IDH2* with *NPM1* may explain the favorable prognosis of R140 *IDH2* mutated AML observed in some studies.

Mutations in *IDH1* and *IDH2* are gain-of-function mutations that result in an NADPH-dependent reduction of α-ketoglutarate to 2-hydroxyglutarate (2-HG); while the *L*-enantiomer of 2-HG is normally produced at low levels physiologically in response to cellular hypoxia, the *R*-enantiomer of 2-HG is an oncometabolite produced specifically in the setting of mutated *IDH* that promotes tumorigenesis [32–34]. R132 mutations in *IDH1*, R172 and R140 mutations in *IDH2* are associated with elevated 2-HG levels in patients with AML [32, 35]. The mutated active site decreases the affinity of IDH for isocitrate and increases its affinity to α-ketoglutarate and NADPH, driving the enzymatic production of 2-HG and decreasing the production of α-ketoglutarate [32].

**Fig. 1:**
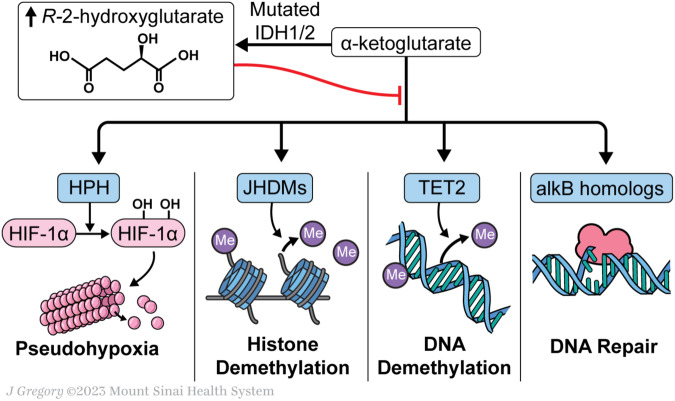
Mutated IDH1/2 converts α-ketoglutarate to *R*-2-hydroxyglutarate, which competitively inhibits α-ketoglutarate dependent enzymes required for DNA and histone demethylation, DNA repair pathways, and HIF-1α hydroxylation and proteasomal degradation. This leads to altered gene expression, chromosomal instability, and persistent pseudohypoxia which drive leukemic transformation. Figure Key: IDH isocitrate dehydrogenase, HIF-1α hypoxia-inducible factor 1α, HPH hypoxia-inducible factor prolyl hydroxylases, JHDM JmjC-domain-containing histone lysine demethylase, TET2 ten-eleven translocation 2, alkB Alkylation B.

2-HG drives oncogenesis through a variety of mechanisms involved in DNA methylation and impaired DNA repair mechanisms. Specifically, 2-HG competitively binds to and inhibits α-ketoglutarate dependent enzymes such as ten-eleven translocation 2 (TET2) [36]. TET2 converts 5-methylcytosines (5-mC) to 5-hydroxymethylcytosine (5-hmC), the active product in the demethylation of cytosine [37]. While physiologic *L*-2-HG levels are too low to compete with α-ketoglutarate for TET2 inhibition, increased *R*-2-HG produced in the setting of mutated *IDH* blocks the function of TET2 and leads to hypermethylation of the HSC genome and impairs differentiation [32, 38]. Secondly, elevated levels of 2-HG cause genetic instability by competitively inhibiting α-ketoglutarate dependent alkB homolog DNA repair enzymes, increasing the potential for malignant transformation [39]. Histone methylation is also increased by 2-HG competitive inhibition of JmjC-domain-containing histone demethylation (JHDM), an α-ketoglutarate dependent enzyme [36]. Histone demethylation is required for progenitor differentiation into terminally differentiated cells. Elevated 2-HG levels have been shown to increase histone methylation which prevent in vitro differentiation of adipocyte cells [40]. Additionally, *R*-2-HG has been shown to induce cytokine independence and block differentiation of HSCs in the presence of growth factors [41]. Mutations in *IDH* have been detected in pre-leukemic cell populations, further suggesting that these effects destabilize the HSC genome and render the cell more susceptible to leukemic transformation, particularly in the context of additional driver mutations [42]. Thus, *IDH* mutations have an oncogenic effect by modifying DNA and histone methylation, altering genetic expression, and inhibiting DNA repair leading to impaired cellular differentiation and dysregulated proliferation (Fig. 1).

In addition to genomic alterations, elevated 2-HG inhibits hypoxia-inducible factor (HIF) prolyl hydroxylases, a group of α-ketoglutarate dependent enzymes that mediate hydroxylation of HIF-1α to target it for proteasomal degradation; *IDH* mutations therefore lead to increased HIF-1α and create a state of persistent pseudohypoxia that promotes tumor growth (Fig. 1) [32, 43, 44]. *IDH* mutations also alter HSC energy catabolism and increase cellular dependence on mitochondrial oxidative phosphorylation. In hypoxic conditions, *IDH1* mutant cells have been shown to increase TCA metabolism and decrease glutamine metabolism, though this effect was not observed in *IDH2* mutant cell lines [45]. Finally, in an *IDH*-mutated glioma model, *R*-2-HG produced by tumor cells was taken up by local T cells in a paracrine fashion and inhibited T cell proliferation and cytokine production, suggesting a role for *IDH* mutations in driving immune dysregulation in the tumor microenvironment, further contributing to oncogenesis [46].

The primary function of the three IDH inhibitors approved for treatment of AML with mutant *IDH* is to limit the production of 2-HG and induce cellular differentiation [47]. This is achieved by reversing the inhibitory effects of 2-HG on DNA methylation, histone methylation and DNA repair, restoring the epigenetic landscape of the HSC. Notably, treatment of a human erythroleukemia cell line TF-1 cells with a cell-permeable form of (*R*)-2HG, meant recapitulate *IDH*-mutant physiology, induces cytokine independent leukemic transformation. This phenotype was shown to be reversible when 2HG is withdrawn, and this effect is inversely related to the duration and dose of 2-HG administered to the cells [48]. IDH inhibitors bind to mutated IDH at an allosteric site, significantly decreasing the production of 2-HG. This concept was first validated in humans in a phase 1 clinical trial of ivosidenib in patients with mutant *IDH1* solid tumors, which showed a 98% decrease in 2-HG levels [49]. Of note, a common toxicity of all IDH inhibitors is the potential for differentiation syndrome, first termed “retinoic acid syndrome,” caused by therapeutic agents that induce rapid differentiation and apoptosis of transformed oncogenic cells [50]. Differentiation occurs at similar rates with both IDH1 and IDH2 inhibitors across clinical trials in *IDH-*mutant AML (Table 1).

**Table 1:** Efficacy and toxicity from clinical trials of currently approved indications for IDH inhibitors and venetoclax in AML.

Phase	Patients	Regimen	Disease status	Primary outcome	Major Toxicities Grade 3 or higher	Ref.
1	179	Ivosidenib 500 mg QD (single arm)	R/R	CR/CRh = 30.4% CR = 21.6% ORR = 41.6% Median time to response = 1.9 months	QTc prolongation (7.8%), differentiation syndrome (3.9%), anemia (2.2%), thrombocytopenia (3.4%), leukocytosis (1.7%)	[65]
1	34	Ivosidenib 500 mg QD (single arm)	ND	CR/CRh = 42.4% CR = 30.3% Median OS = 12.6 months	Differentiation syndrome (9%), anemia (12%), thrombocytopenia (15%)	[66]
3	146	Azacitidine 75 mg/m^2^ + ivosidenib 500 mg QD versus placebo	ND	EFS = 37% vs 12%	Differentiation syndrome (4% vs 4%), febrile neutropenia (28% vs 34%), infection (21% vs 30%)	[68]
1/2	153 (expansion phase)	olutasidenib 150 mg BID (single arm)	R/R	CR/CRh = 35% Median duration CR/CRh = 25.9 months Median duration of response = 11.6 months	Differentiation syndrome (9%) Febrile neutropenia (20%), thrombocytopenia (16%), anemia (20%)	[77]
1/2	126 (expansion phase)	Enasidenib 100 mg QD (expansion dose, single arm)	R/R	Expansion phase: CR = 20.2% ORR = 38.5% Median OS = 9.3 months	Expansion phase: Hyperbilirubinemia (8%), differentiation syndrome (7%)	[80]
3	319	Enasidenib 100 mg QD versus conventional care	R/R	CR = 23.4% vs 3.7%, CR/CRi/CRp =29.7% vs 6.2%, ORR = 40.5% vs 9.9%,	Differentiation syndrome (5.1% vs 0%), hyperbilirubinemia (10.8% vs 0%)	[82]
3	498	Azacitidine 75 mg/m^2^ + venetoclax 400 mg QD versus placebo	ND	m*IDH* CR/CRi =79.0% *IDH* wild-type CR/CRi = 62.2% m*IDH* median duration of response =17.5 months Wildtype median duration of response = 29.5 months	Febrile neutropenia (42% vs 19%), neutropenia (42% vs 28%) anemia (26% vs 20%), thrombocytopenia (45% vs 38%)	[61]
3	211	Low-dose cytarabine 20 mg/m^2^ + venetoclax 600 mg QD versus placebo	ND	m*IDH* CR/CRi = 57% Full cohort CR/CRi = 48% m*IDH* OS = 19.4 months Full cohort OS = 10.1 months	Febrile neutropenia (32% vs 29%), neutropenia (47% vs 16%), anemia (25% vs 22%) thrombocytopenia (45% vs 37%)	[63]

*CR* complete response, *CRh* CR with partial hematologic recovery, *ORR* overall response rate, *OS* overall survival, *CRi* CR with incomplete hematologic recovery, *CRp* CR with incomplete platelet recovery, *EFS* event-free survival, QD once daily, *BID* twice daily, R/R relapse/refractory, *ND* newly diagnosed.

Differentiation syndrome (DS) is a serious adverse event that may be fatal if not recognized and treated in a timely manner. Primary symptoms are fever and/or respiratory distress, along with a chest X ray demonstrating pulmonary infiltrates [50, 51]. Additional symptoms include weight gain, peripheral edema, pleuro-pericardial effusions, hypotension and acute renal failure [50, 51]. Although a different disease, we draw a significant portion of our knowledge of differentiation syndrome from our experience in managing acute promyelocytic leukemia (APL). In a large study of 739 patients with APL treated with all-*trans* retinoic (ATRA) acid plus idarubicin classified DS as two or more of the previously mentioned symptoms [51]. The study further classified DS as moderate when 2–3 symptoms are present and severe with 4 or more symptoms [51].

As mentioned, our understanding of the pathogenesis of DS is largely drawn from studies of APL that demonstrate ATRA and arsenic trioxide (ATO) induce the maturation of promyelocytes and activates a systemic inflammatory response associated with increased cytokine production, endothelial damage with capillary leak, occlusion of the microcirculation and tissue infiltration [52, 53]. Rapidly differentiating myeloid cells release inflammatory cytokines, notably interleukin (IL)-1, IL-6, IL-8, and tumor necrosis factor alpha, leading to inflammation, and end organ damage [53].

The mainstay of management involves respiratory support and corticosteroids, typically with dexamethasone and should be initiated promptly on suspicion. Dose recommendation is 10 mg twice daily by intravenous access until symptom resolution followed by a steroid taper. In severe cases of DS, clinicians should consider holding IDH-inhibitor therapy until clinical recovery [54, 55].

## BCL2 inhibition in *IDH*-mutated AML

The anti-apoptotic B-cell lymphoma protein 2 (Bcl-2) prevents programmed cellular apoptosis by stabilizing the mitochondrial membrane and preventing the release of cytochrome c and caspase activation in response to oxidative stress or DNA damage [56]. Bcl-2 overexpression in AML is associated with chemotherapy resistance and reduced survival [57–59]. Notably, 2-HG inhibits cytochrome oxidase in the ETC and lowers the threshold for release of cytochrome-c from the mitochondrial membrane, triggering apoptosis. *IDH*-mutated cells are, therefore, more dependent on Bcl-2 for maintenance of mitochondrial stability and thus are more susceptible to Bcl-2 inhibition [60]. Given the decreased threshold for mitochondrial depolarization and increased reliance on Bcl-2 to prevent cell death, venetoclax-based regimens represent a rational strategy for treatment of *IDH*-mutated AML.

The VIALE-A phase 3 clinical trial established azacitidine plus venetoclax, a Bcl-2 inhibitor, as standard of care for newly diagnosed AML patients unfit for standard induction therapy [61]. In the subgroup analysis, patients with *IDH* mutations demonstrate a more pronounced responses with the addition of venetoclax compared to *IDH* wild-type patients [62]. *IDH*-mutated patients had a composite complete response (CR) plus CR with incomplete count recovery (CRi) rate of 79.0% with azacitidine plus venetoclax versus 62.6% in *IDH* wild-type patients, and median duration of response of 29.5 months versus 17.5 months in *IDH* mutated and wild-type patients, respectively [62].

The VIALE-C trial was a similar phase 3 study of 211 patients with newly diagnosed AML ineligible for standard induction therapy who were randomized to receive low-dose cytarabine (LDAC) plus venetoclax or placebo [63]. In the subgroup analysis, patients with *IDH1/2* mutated AML had a longer median overall survival (OS) of 19.4 months versus 10.1 months in the overall study cohort. The composite of CR/CRi with the addition of venetoclax was 57% in *IDH1/2* mutated AML versus 48% in the overall population [63]. Based on these data, inhibition of Bcl-2, in combination with hypomethylating agent azacitidine, represent an effective treatment modality in newly diagnosed *IDH*-mutated AML.

## Targeting IDH1 - ivosidenib

Ivosidenib (AG-120), is a highly selective, reversible, small molecule inhibitor of mutant IDH1 and was the first Food and Drug Administration (FDA)-approved targeted therapy in its class. It is an allosteric inhibitor that has been shown to significantly reduce 2-HG levels in pre-clinical models and induce cellular differentiation in primary patient AML samples ex vivo [64].

The FDA accelerated approval for ivosidenib in 2018 was based on an open-label, single arm, multicenter phase 1 dose escalation and expansion study of ivosidenib in 179 patients with R/R AML with a confirmed *IDH1* mutation [65]. Ivosidenib 500 mg daily was administered orally until disease progression or unacceptable toxicity, and key efficacy endpoints were CR rate, composite CR plus complete response with partial hematologic recovery (CRh), duration of response, and the rate of transfusion independence. Treatment was generally well tolerated, with low rates of grade 3 and above adverse events including QTc prolongation (7.8%), differentiation syndrome (3.9%), anemia (2.2%), thrombocytopenia (3.4%) and leukocytosis (1.7%). Efficacy was assessed in 125 patients, who demonstrated a composite CR/CRh of 30.4% (95% confidence interval [CI], 22.5–39.3), including CR of 21.6% (95% CI, 14.7–29.8), overall response rate (ORR) of 41.6% (95% CI, 32.9–50.8), and median time to response of 1.9 months (range 0.8–4.7). The median durations of CR/CRh, CR, and overall response were 8.2 months (95% CI, 5.5–12.0), 9.3 months (95% CI, 5.6–18.3), and 6.5 months (95% CI, 4.6–9.3), respectively. Additionally, 29/84 patients (35%) who were previously transfusion dependent developed transfusion independence [65].

Notably, clinical outcomes with ivosidenib were associated with molecular responses. The *IDH1* mutation variant allele frequency (VAF) decreased over time in patients with a best response of CR/CRh but remained stably elevated in patients who did not achieve CR/CRh. Further, *IDH1* mutation clearance was significantly associated with a best response of CR/CRh (p = 0.003); whereas 21% (7/34) of patients with CR/CRh exhibited *IDH1* mutation clearance from bone marrow mononuclear cells, all patients who did not achieve CR/CRh with available data maintained *mIDH1* status [65].

Ivosidenib was also evaluated in the up-front setting in *IDH1*-mutated AML patients ineligible for standard induction therapy. A subgroup of 34 newly diagnosed patients from the phase 1 study above were treated with ivosidenib 500 mg daily as monotherapy [66]. Differentiation syndrome occurred in 18% of patients, including 9% with grade 3 or higher events. Patients demonstrated a CR of 30.3% (95% CI, 15.6–48.7), composite CR/CRh of 42.4% (95% CI, 25.5–60.8), and median OS of 12.6 months (95% CI, 4.5–25.7) [66]. A subsequent phase 1b clinical trial enrolled 23 newly diagnosed patients and treated them with ivosidenib 500 mg daily with subcutaneous azacitidine 75 mg/m^2^ on days 1–7 of a 28-day cycle. The ORR was 78.3% (95% CI, 56.3–92.5), and the CR rate was 60.9% (95% CI, 38.5–80.3) [67].

These data led to the phase 3 AGILE study, in which 146 patients with newly diagnosed, *IDH1-*mutated AML ineligible for induction therapy were randomized to oral ivosidenib 500 mg daily or placebo plus subcutaneous azacitidine 75 mg/m^2^ on days 1–7 in a 28-day cycle [68]. The primary endpoint was event-free survival (EFS). Ivosidenib had a favorable toxicity profile compared to the placebo group, including similar rates of grade 3 or above adverse events including febrile neutropenia (28% vs 34%), neutropenia (27% vs 16%), bleeding events (6% vs 7%), infection (21% vs 30%) and differentiation syndrome (4% vs 4%). The ivosidenib group had a 12-month EFS of 37% compared to 12% in the placebo group, and CR at 24 weeks was 38% compared to 11%. The median OS was 24 months (95% CI, 11.3–34.1) in treatment group versus 7.9 months (95% CI, 4.1–11.3) in the control arm (*p* = 0.001) [68]. However, only 2 patients in the placebo arm of the study had access to ivosidenib at progression, which limits the interpretation of survival analysis in a population that would otherwise receive ivosidenib in the refractory setting, as this was already established as standard of care in the United States [69]. Therefore, true impact on OS in the front line vs. second line setting remains unknown.

Another limitation of the AGILE trial has been the use of azacitidine alone as a control group. Azacitidine with venetoclax became the accepted standard of care therapy during the execution of the AGILE study. This is particularly important to note given the promising efficacy of venetoclax in the *IDH*-mutated subgroup of the VIALE-A trial noted above [61, 62]. While retrospective experience presented at the American Society of Hematology (ASH) 2023 annual meeting showed favorable outcomes with azacytidine plus ivosidenib compared to azacytidine plus venetoclax, randomized data are lacking [70]. Further study is also needed to determine the role of ivosidenib in addition to the combination of hypomethylating agents and venetoclax.

Several studies combining IDH inhibitors and venetoclax-based regimens have been conducted and are ongoing. To evaluate the efficacy of ivosidenib with venetoclax-based regimens, 31 patients with *IDH1*-mutated AML were studied in a phase 1b trial of ivosidenib and venetoclax with or without azacytidine [71]. The composite CR rate was 90% with the addition of azacitidine compared to 83% with ivosidenib and venetoclax alone. In the overall study population, median OS was 42 months (95% CI, 42 to not reached) and median EFS was 36 months (95% CI, 23 to not reached), and 63% of the 16 evaluable patients attained measurable residual disease (MRD)-negative remission. Maximum tolerated dose was not reached, with most common grade 3 and above adverse events including febrile neutropenia (29%), pulmonary infection (19%), and differentiation syndrome (10%) [71]. A phase 2 study is currently underway (NCT03471260). Preliminary data of a phase 1b/2 trial of triplet therapy with decitabine/cedazuridine, venetoclax, and ivosidenib showed encouraging response rates and tolerability in newly diagnosed and relapsed patient populations [72].

Ivosidenib has additionally been studied in patients fit for intensive chemotherapy. In a phase 1 study, 60 patients with *IDH1*-mutated AML received ivosidenib with cytarabine and anthracycline (7 + 3) induction and consolidation, followed by maintenance ivosidenib until progression or allogeneic stem cell transplant [73]. Treated patients showed a composite CR rate of 72%, 12-month OS of 78%, and median OS not yet reached at median follow up of 9.3 months. Time to count recovery and adverse events were similar to historical controls receiving 7 + 3 induction, with low rates of differentiation syndrome given concurrent cytotoxic therapy [73]. A phase 3 randomized study of ivosidenib with intensive chemotherapy in newly diagnosed *IDH1*-mutated AML is currently underway (NCT03839771) It is important to note that given the design of this study, which plans to add ivosidenib to induction, consolidation, and as a maintenance therapy, it may be challenging to understand the relative impact of ivosidenib treatment within each treatment stage. That is, if there is a significant benefit observed in the study, is that benefit derived from the combination of ivosidenib with induction chemotherapy or is the benefit largely derived from post-induction maintenance.

Given the concurrent approval of ivosidenib for both up-front and salvage therapy, further study is needed to elucidate the optimal sequencing of therapy to improve survival. Additional open questions to be evaluated include the role of triple therapy with azacitidine and venetoclax, combinations with venetoclax-based versus intensive chemotherapy regimens in fit patients, and combination therapy approach in R/R patients. Other trials currently under investigation include ivosidenib with CPX-351 in either newly diagnosed or R/R disease (NCT04493164), ivosidenib with FLAG salvage therapy in R/R AML (NCT04250051), decitabine/cedazuridine and venetoclax with ivosidenib in R/R patients (NCT04774393), and a study of sequential therapy with azacitidine/venetoclax and azacitidine/ivosidenib (NCT05401097).

## Targeting IDH1 - olutasidenib

Olutasidenib (FT-2102) is the most recently FDA-approved selective inhibitor of mutant IDH1 for R/R AML. Olutasidenib is highly selective, potent, orally administered, and penetrates the blood-brain-barrier, with no impact on wild-type IDH1 function [74]. Olutasidenib is a quinolone derived allosteric inhibitor of mutant IDH1 that binds to a hydrophobic pocket near the active site and stabilizes mutant IDH1 in an open, inactive state, preventing the conformational change necessary to produce 2-HG [74]. Unlike ivosidenib, it binds mutated IDH1 in a 2:1 stoichiometric ratio, which theoretically offers potential to overcome certain ivosidenib resistance mutations [75]. In *IDH1*-mutant xenograft tumor models, olutasidenib potently suppressed 2-HG production and induced differentiation of leukemia cells [74].

Olutasidenib is currently being investigated in a phase 1/2 open label, multicenter study in R/R or treatment naive *IDH1*-mutated AML or MDS patients, either as monotherapy or in combination with azacitidine (NCT02719574). In the phase 1 study evaluating safety and toxicity, 32 patients received olutasidenib monotherapy (26 with AML), and 46 patients received combination therapy with azacitidine (39 with AML) [76]. A dose escalation of 150 mg once daily, 300 mg once daily, and 150 mg twice daily had no dose-limiting toxicities, with 150 mg twice daily showing greatest reduction in 2-HG levels. Grade 3–4 differentiation syndrome occurred in 13% of patients in both arms but resolved with either steroids, supportive care, or dose interruption without recurrence. Combination therapy and monotherapy had similar rates of grade 3–4 febrile neutropenia (28% vs 22%), thrombocytopenia (41% vs 28%), and anemia (20% vs 22%), with combination therapy showing increased rates of grade 3–4 neutropenia (28% vs 6%), fatigue (17% vs 6%), nausea (9% vs 0%), and QTc prolongation (7% vs 0%). In the AML subgroup, ORR was 38% for monotherapy and 56% for combination therapy, with composite CR rates of 27% and 28%, respectively [76].

In a pre-planned interim analysis of the phase 2 component of the study, 153 IDH1 inhibitor naive patients with R/R *IDH1*-mutant AML were treated with olutasidenib monotherapy at a dose of 150 mg twice daily for continuous 28-day cycles [77]. Most patients received prior induction chemotherapy (97%) and consolidation cytarabine (71%), with a median of 2 prior regimens. The primary endpoint was a composite of CR/CRh, with secondary endpoints including ORR, duration of CR/CRh, duration of overall response, and rate of transfusion independence. The composite CR/CRh rate was 35% (95% CI, 27–43) with an ORR of 48% (95% CI, 40 to 56.7), and CR/CRh rates were similar regardless of prior venetoclax exposure (33% vs 35%). Patients who developed CR/CRh had early responses, with median time to CR/CRh of 1.9 months and had durable responses with median duration of 25.9 months. The median OS was 11.6 months (95% CI, 8.9 to 15.5), with overall median duration of response of 11.7 months. In patients who did not respond, the OS was 4 months. Of 86 patients who were transfusion dependent at baseline, the 56-day transfusion independence rate was achieved in 29 (34%) of those patients in all response groups. The toxicity profile was similar to the phase 1 data, with grade 3 and above adverse events including febrile neutropenia (20%), anemia (20%), thrombocytopenia (16%), and neutropenia (13%), as well as reversible liver enzyme elevations in 25% and transient QTc prolongation in 8% of patients. Differentiation syndrome occurred in 14% of patients overall, 9% grade 3 and above, primarily occurring in the first 2 cycles, and caused one patient death [77].

Olutasidenib performed favorably in R/R AML compared to historical controls and showed similar rates of composite CR, but longer duration of response compared to the analogous ivosidenib trial. However, the two drugs have not been compared head-to-head to inform the choice of a first line IDH1 inhibitor in this population. Additionally, the interim phase 2 olutasidenib analysis excluded patients with prior IDH-inhibitor exposure, and it has not been formally tested as a salvage therapy in patients failing another IDH1 inhibitor, though a small initial subgroup analysis suggested the possibility of responses after ivosidenib failure [78]. Further trials are needed to evaluate olutasidenib combination therapies, particularly with venetoclax-based regimens, and to optimize sequencing of IDH1 inhibitors in both newly diagnosed and R/R patients.

## Targeting IDH2 – enasidenib

Enasidenib (AG-221) is a first-in-class highly selective oral IDH2 inhibitor that is now FDA-approved for *IDH2*-mutated R/R AML. In preclinical ex vivo and xenograft models, enasidenib has been shown to allosterically inhibit IDH2, suppress 2-HG production, and induce cellular differentiation in primary human *IDH2*-mutant AML cells [79].

Enasidenib was approved as monotherapy for R/R AML based on a phase 1/2 dose escalation and expansion study of 239 patients with *IDH2-*mutated AML, including 176 with R/R disease and 63 who were treatment naïve [80]. In the dose-escalation phase, 113 subjects were enrolled at 13 doses ranging from 50 to 650 mg per day, with maximum tolerated dose not reached, and 126 patients were entered into the dose expansion cohort, with a dose of 100 mg daily chosen based on pharmacokinetics and 2-HG suppression. Enasidenib was well tolerated, with most common grade 3/4 adverse events including hyperbilirubinemia (12%) and differentiation syndrome (6%) in the overall cohort. In the efficacy analysis, 109 patients with R/R AML were analyzed at the target dose of 100 mg daily. The CR rate was 20.2% (95% Cl, 13.1–28.9) and ORR was 38.5% (95% CI, 29.4–48.3), with median time to response of 1.9 months (range, 0.5–9.4 months). The median OS in R/R patients was 9.3 months (95% CI, 8.2–10.9) overall and 19.7 months (95% CI, 11.6 to not reached) in those with CR, median EFS was 6.4 months (95% CI, 5.4 to 7.5), and median duration of response 5.6 months (95% CI, 3.8–9.7) [80].

In a biomarker analysis, potent 2-HG suppression was observed in all patients receiving enasidenib, although the degree of 2-HG suppression alone did not correlate with or predict clinical response [81]. As seen in prior preclinical models, response to enasidenib was mediated by cellular differentiation as opposed to direct cellular cytotoxicity. Additionally, patients who did not respond to therapy were found to have co-occurring oncogenic mutations in the NRAS or MAPK pathways, suggesting that clonal evolution and upregulation of parallel growth pathways may mediate enasidenib resistance [81].

Based on these promising clinical results, a phase 3 randomized international trial evaluated enasidenib monotherapy versus conventional care regimens (CCR) in 319 patients older than 60 years with R/R *IDH2*-mutated AML [82]. Patients were pre-selected to CCR of azacitidine, intermediate-dose cytarabine, low-dose cytarabine, or supportive care and then randomized 1:1 to enasidenib 100 mg daily or CCR. Enasidenib showed a comparable safety profile to CCR, with notable differences including the presence of differentiation syndrome (14% overall, 5.1% grade 3 and above), which required treatment with steroids in the majority of cases, and increased hyperbilirubinemia (19.7% vs 0.7% overall, 10.8% vs 0% grade 3). The enasidenib group showed significantly increased rates of CR (23.4% vs 3.7%, *p* < 0.001), composite CR/CRi/CR with incomplete platelet recovery (CRp) (29.7% vs 6.2%, *p* < 0.001), and ORR (40.5% vs 9.9%, *p* < 0.001) compared to the CCR group. Enasidenib additionally showed significantly prolonged median EFS of 4.9 versus 2.6 months (HR 0.68, 95% CI, 0.52–0.91, *p* = 0.008). However, despite improved responses, there was no significant difference in median OS of 6.5 months with enasidenib versus 6.2 months for CCR (HR 0.86, 95% CI, 0.67–1.10, *p* = 0.23). There was a signal towards increased one-year survival with enasidenib of 37.5% versus 26.1% (Δ+11%; 95% CI, 1–22), which was particularly prominent in patients with *IDH2* R172 and poor risk cytogenetics [82]. Nonetheless, the lack of OS benefit with enasidenib despite the use of a suboptimal control arm without the inclusion of venetoclax raises concern for the role of enasidenib monotherapy in R/R AML. Enasidenib is currently being evaluated as combination therapy for R/R *IDH2*-mutated AML in phase 1b/2 studies with venetoclax (NCT04092179) and decitabine/cedazuridine with venetoclax (NCT04774393), with preliminary data presented at ASH 2023 showing feasibility and favorable outcomes with both combinations [72, 83].

In newly diagnosed *IDH2*-mutated AML, enasidenib was initially studied as monotherapy. In a multicenter, open-label, phase 1/2 trial (AG221-C-001), 39 patients deemed unfit for intensive chemotherapy were treated with daily enasidenib for 28-day cycles [84]. In the dose escalation portion, 10 patients were treated with doses ranging from 50 to 450 mg daily, followed by a dose expansion cohort at 100 mg daily. Grade 3 and above adverse events included cytopenias (21%), hyperbilirubinemia (13%), and differentiation syndrome (10%). The ORR was 30.8% (95% CI, 17.0–47.6) with a CR of 18%, with median duration of response not reached at median follow up of 8.4 months. The median OS was 11.3 months (95% CI, 5.7–15.1) [84]. This trial demonstrates safety and tolerability of enasidenib with evidence of disease-targeting activity in *IDH2*-mutated AML.

Based on preclinical data showing synergy with hypomethylating agents in inducing differentiation, enasidenib was evaluated in newly diagnosed patients in combination with azacitidine. In a phase 1b/2 clinical trial, 101 patients with newly diagnosed *IDH2*-mutated AML ineligible for induction therapy were randomized 2:1 to azacitidine 75 mg/m^2^ (days 1–7 of a 28-day cycle) plus enasidenib 100 mg daily (*n* = 68) versus azacitidine alone (*n* = 33) [85]. The enasidenib treatment group had higher rates of grade 3/4 differentiation syndrome (10% vs 0%), thrombocytopenia (37% vs 19%), and neutropenia (37% vs 25%), although with similar rates of febrile neutropenia (16% vs 16%). The enasidenib treatment group had significantly higher rates of ORR (74% vs 36%, *p* = 0.0003), CR (54% vs 12%, *p* < 0.0001), and composite CR/CRp (57% vs 18%, *p* = 0.0002), as well as longer median duration of response (24.1 vs 9.9 months). Significant reduction in baseline 2-HG levels in the enasidenib group was again noted regardless of clinical response. Enasidenib had a trend toward longer median EFS (15.9 vs 11.9 months, *p* = 0.11), but there was no difference in median OS (22.0 vs 22.3 months, *p* = 0.97), though patients in the combination therapy group who achieved a CR had a median OS that was not yet reached [85]. Of note, about one-third of patients in the control group received enasidenib at progression as post-protocol therapy, which confounds survival analysis and may explain why the EFS trend did not translate into an OS benefit.

A major limitation of the azacitidine/enasidenib study is, again, the comparison to a control arm lacking venetoclax. In a small preliminary analysis of a phase 2 study of azacitidine and enasidenib that allowed concomitant venetoclax (NCT03683433), patients receiving triplet therapy in either newly diagnosed (*n* = 4) or R/R (*n* = 7) disease had CR rates of 100% and 86%, respectively, with median EFS and OS not reached at follow up of 11.2 months [86]. Further randomized data are needed to assess azacitidine and enasidenib compared to or in combination with venetoclax in newly diagnosed patients unfit for standard induction therapy. As above, a phase 1b/2 study of enasidenib with decitabine/cedazuridine and venetoclax is ongoing, with promising preliminary data in newly diagnosed patients [72].

In patients eligible for intensive therapy, enasidenib was evaluated in a phase 1 study of 91 patients with newly diagnosed *IDH2*-mutated AML [73]. Patients received enasidenib 100 mg daily along with 7 + 3 induction and consolidation, with an option for subsequent maintenance enasidenib until progression or allogeneic stem cell transplant. The most common grade 3 or above adverse events during induction were hyperbilirubinemia (16%), rash (14%), and hypophosphatemia (13%), without significant differentiation syndrome. Patients demonstrated a CR of 55%, composite CR/CRi/CRp of 74%, and median OS of 25.6 months [73]. A phase 3 randomized study of standard induction chemotherapy with enasidenib is ongoing (NCT03839771).

In summary, while enasidenib is approved for R/R *IDH2*-mutated AML, randomized phase 3 data did not reproduce a survival benefit compared to conventional therapy. Results in newly diagnosed patients have been similarly mixed: azacitidine/enasidenib showed EFS benefit without a difference in OS, and enasidenib with a conventional induction regimen showed promise in the early phase, with randomized data forthcoming. Ongoing trials are needed for combination regimens, particularly with venetoclax, to better delineate the optimal role and sequencing for enasidenib in AML-directed therapy.

## Conclusion

AML is a heterogenous disease with poor prognosis, and targeted approaches based on individualized mutational profiling may improve survival. In particular, IDH inhibitors are an important therapeutic option for *IDH*-mutated patients ineligible for intensive induction or with R/R disease, populations with historically poor outcomes. There are ongoing clinical trials to determine the best use of these novel agents in both the front line and relapsed setting, with ongoing questions including IDH inhibitors with venetoclax in combination therapies, the incorporation of IDH inhibitors into intensive cytotoxic regimens for both induction and salvage therapy, and sequencing of therapy in *IDH1*-mutated patients eligible for both ivosidenib and olutasidenib. Additionally, efficacy data in phase 3 study cohorts are often driven by a subset of patients with durable responses to therapy and prolonged survival. This highlights the potential potency of IDH inhibitors but also challenges clinicians to better understand mechanisms of resistance and identify predictors of response to implement these agents more effectively into clinical practice. IDH inhibitors represent a promising avenue to provide personalized care and continue to improve outcomes for patients with AML.

## Data Availability

All data discussed within the manuscript are available within original research cited.
